# Evaluation of the measurement properties of the Manchester foot pain and disability index

**DOI:** 10.1186/1471-2474-15-276

**Published:** 2014-08-12

**Authors:** Babette C van der Zwaard, Caroline B Terwee, Edward Roddy, Berend Terluin, Henriette E van der Horst, Petra JM Elders

**Affiliations:** EMGO + Institute for health and care research, Department of general practice and elderly care medicine, VU University Medical Centre, Amsterdam, The Netherlands; EMGO + Institute for health and care research, Department of Epidemiology and Biostatistics, VU University Medical Centre, Amsterdam, The Netherlands; Research Institute for Primary Care and Health Sciences, Keele University, Staffordshire, UK

## Abstract

**Background:**

The Manchester Foot Pain and Disability Index (MFPDI, 19 items) was developed to measure functional limitations, pain and appearance for patients with foot pain and is frequently used in both observational studies and randomised controlled trials. A Dutch version of the MFPDI was developed. The aims of this study were to evaluate all the measurement properties for the Dutch version of the MFPDI and to evaluate comparability to the original version.

**Method:**

The MFPDI was translated into Dutch using a forward/backward translation process. The dimensionality was evaluated using exploratory and confirmatory factor analysis. Measurement properties were evaluated per subscale according to the COSMIN taxonomy consisting of: reliability (internal consistency, test-retest reliability and measurement error), validity (structural validity, content validity and cross-cultural validity comparing the Dutch version to the English version) responsiveness and interpretation.

**Results:**

The questionnaire consists of three scales, measuring foot function, foot pain and perception. The reliability of the foot function scale is acceptable (Cronbach’s α > 0.7, ICC = 0.7, SEM = 2.2 on 0-18 scale). The construct validity of the function and pain scale was confirmed and only the pain scale contains one item with differential item functioning (DIF). The responsiveness of the function and pain scale is moderate when compared to anchor questions.

**Conclusion:**

Results using the Dutch MFPDI version can be compared to results using the original version. The foot function sub-scale (items 1-9) is a reliable and valid sub-scale. This study indicates that the use of the MFPDI as a longitudinal instrument might be problematic for measuring change in musculoskeletal foot pain due to moderate responsiveness.

## Background

Foot pain is common in older people and is associated with functional limitations in foot related activities; prevalences between 14.9 and 41.9% have been reported in people over 50 years [1–6]. The Manchester Foot Pain and Disability Index (MFPDI) is a 19 item tool developed to measure foot pain and foot related function in patients with foot pain [7]. It intends to measure 3 constructs: functional limitation, pain and personal appearance [7]. Statements relating to the 3 constructs can be answered in terms of frequencies: ‘none of the time’, ‘on some days’ and ‘on most/every day(s)’. Previous studies have evaluated some of the measurement properties of the questionnaire. Internal consistency (IC) is the most evaluated property [7–12] although some studies evaluated it over the entire questionnaire instead of over the three constructs (i.e. sub-scales) [7, 9, 10]. The studies that did evaluate each sub-scale separately have found the scales to be internal consistent [8, 12]. Test-retest reliability has been analysed by Roddy et al. [12] and found to be moderate for the pain and the appearance scale and both moderate and acceptable for the functional limitations scale depending on the inclusion criterion. Construct validity has been described in previous studies although the amount of hypotheses tested was small in one study [7] and no hypotheses were stated *a priori* in another [10]. The characteristics of the items in the questionnaire have been thoroughly examined using item response theory testing (IRT). The MFPDI consists of items that allow severe cases to be distinguished from the less severe cases [8, 11].

The MFPDI is not merely a descriptive tool (e.g. in cross-sectional studies) but it is also used as a tool to measure change over time as a result of an intervention [13–15]. When interpreting change scores in a randomised controlled trial (RCT) or another longitudinal study, it is vital to have knowledge about measurement properties like measurement error, responsiveness and interpretation values like minimal important change (MIC) and smallest detectable change (SDC). Currently these properties have not been assessed yet. For the purpose of a Dutch study on forefoot pain in people 50 years of age or older the MFPDI had to be translated into Dutch [15]. The aims of the current study are, firstly, to create a Dutch version of the MFPDI. Secondly, to evaluate measurement properties based on the Classical Test Theory (CTT), including a cross-cultural validation of the Manchester Foot Pain and Disability Index using the Dutch translated version.

## Method

This study describes an evaluation of measurement properties of the MFPDI starting with the translation of the questionnaire into Dutch. The Medical Ethics Committee of the VU University Medical Centre in Amsterdam has approved the design of this study (No. 2009/267).

### Translation

A forward-backward method of translation was used to obtain a Dutch version of the MFPDI [16]. Two translators; a general practitioner and a physical education teacher, both with additional experience with English, translated the original version into Dutch. During the consensus meeting with the two translators and guided by PE and BvdZ, a consented Dutch version was established. This version was then translated back into English by two native speakers; a UK scientific researcher and a UK physician assistant. Differences between the original and the translated version were discussed by BvdZ and PE and the Dutch version was accordingly adapted. The Dutch version was then tested by 10 participants, who were asked about intelligibility and completeness of the list. Concerns identified regarding intelligibility and completeness were discussed between BvdZ and PE and adapted if required.

### Participants

As part of an RCT that compares treatment of forefoot pain by means of podiatric care or shoe advice, participants aged 50 years and over with non traumatic forefoot pain that lead to functional limitations were recruited [15]. Exclusion criteria were: the presence of diabetic neuropathy, non musculoskeletal foot pain (e.g. warts) or pain caused by rheumatoid arthritis. Participants had to be able to walk un-assisted for 7 metres. These participants completed the MFPDI on multiple occasions during the trial as part of self administered questionnaires. The MFPDI was used as a screening tool; participants had to score at least one item on the MFPDI as occurring ‘on most/every day(s)’ to be considered limited functionally and thus eligible for the trial.

### Procedure and outcome measures

Preceding inclusion the MFPDI was used as a screening tool (NL Ts). One to three weeks later the participants were included in the trial and completed a comprehensive questionnaire with the below mentioned comparator instruments and the MFPDI as a baseline measurement (NL T0). Since all participants had at least 3 months of foot pain and were considered to have limited functionality indicating a stable state no additional measures were taken to assess possible changes between baseline and screening. Three months after baseline a similar questionnaire was completed again (NL T3). The lowest response in the MFPDI was marked as 0 and the highest response as 2. The COSMIN taxonomy was utilized to evaluate every measurement property of the MFPDI questionnaire [17, 18].

As comparator instruments to the MFPDI several other questionnaires were completed: the Foot Function Index-5 pt [19] (FFI-5 pt), SF12® and an 11 point pain intensity numeric rating scale (NRS where 0 = no pain and 10 = worst possible pain). The FFI-5 pt is a questionnaire on foot function, consisting of two subscales: foot pain and foot related activities, consisting of respectively 7 and 8 items. The original FFI is more extensive (23 items), has response options using visual analogue scales (VAS) and was developed for people with rheumatoid arthritis. The FFI-5 pt has been validated for use in the elderly with non rheumatic foot problems, has 5 response options (ranging from "no pain" to "intense pain" and from "no difficulty" to "impossible") and has already been translated into Dutch [19]. In addition to the comparator instruments and the MFPDI, two global perceived effect (GPE) questions on foot pain and foot related disability were added to the NL T3 questionnaire: "How would you judge the pain in your foot now, compared to three months ago?" and "How would you judge the performance of foot related activities now, compared to three months ago?". These GPE questions could be answered with: "much worse, worse, no change, better or much better".

### UK sample for cross-cultural validation

For the purpose of assessing cross-cultural validity, a sub-sample of data was obtained from two phases of the North Staffordshire Osteoarthritis Project (NorStOP) [20–22]. The sampling frame consisted of all adults aged 50 years and over registered with six general practices in North Staffordshire, UK. Briefly, NorStOP consisted of a two-stage cross-sectional questionnaire. Stage 1 consisted of a postal Health Survey questionnaire. Respondents to this questionnaire who reported experiencing foot pain in the last 12 months were sent a Regional Pain Survey questionnaire which contained the MFPDI. Respondents were also asked to provide consent for review of their medical records.

Criteria for inclusion in the sub-sample for this analysis were: having foot pain in the last 12 months, reporting at least 2 items on the MFPDI occurring on "some days" or "most/every day(s)", and both providing consent to medical record review and consulting their GP with musculoskeletal foot problems in the 18 months prior to the baseline Health Survey. Participants who had diabetes mellitus (self-report in Health Survey questionnaire) or had consulted their GP for rheumatoid arthritis in the 18 months prior to the baseline Health Survey were excluded.

### Statistical analyses

First, the dimensionality of the Dutch version of the MFPDI was investigated using an exploratory factor analysis (EFA). A confirmatory factor analysis (CFA) was used to evaluate the fit of the 3 construct conceptual model [7, 11, 12] on the Dutch data. CFA was also used to test both the 3 construct conceptual model and the EFA findings on the UK data. The last two items of the MFPDI ("I am unable to carry out my previous work" and "I no longer do all my previous activities") were not applicable for a large number of participants (39%) and were thus not used for either analyses. Model fit was evaluated by comparing Root Mean Square Error of Approximation (RMSEA), Comparative Fit Index (CFI) and Tucker-Lewis fit Index (TLI) from the two different models. RMSEA ≤0.06 indicated good model fit and for CFI and TLI a cut off value of 0.95 was chosen [23, 24].

#### Reliability

**Internal consistency (IC)** IC was evaluated per sub-scale of the NL T0 data using Cronbach’s α. An outcome between 0.7-0.95 was considered acceptable [25].

### Test-retest reliability

To evaluate test-retest reliability, the intra class correlation (ICC_agreement_) was calculated comparing the NL Ts to the NL T0 data. Variance components were estimated using the VARCOMP tool in SPSS. The ICC_agreement_ was calculated by dividing the variance between patients by the sum of the variance between patients ( ), the variance due to systematic differences between observations ( ) and the residual variance ( ) (equation 1) [26]. An ICC of 0.7 or higher was deemed acceptable [25].
1

### Measurement error

The standard error of measurement (SEM_agreement_) was calculated using the same variance components used for the ICC_agreement_ calculation (equation 2) [26], comparing the NL T0 with the NL Ts data.
2

#### Validity

**Construct validity** In order to evaluate construct validity the ‘hypotheses testing’ method was chosen in absence of a gold standard [27]. Hypotheses were formulated based on two general assumptions [27]. First, correlations between MFPDI subscales and (subscales of) similar questionnaires should be >0.50. Second, correlations between MFPDI subscales and dissimilar questionnaires or subscales should be lower. The following seven *a priori* defined hypotheses stating expected correlations between sub-scales of the MFPDI and the sub-scales of the FFI-5pts, SF-12 and NRS within the NL T0 data were tested using Pearson correlations:

1. The score of the MFPDI- function items (MFPDI-f) correlates with the score of the FFI- 5pts function items (FFI-f) with R > 0.5;
2. The score of the MFPDI-f correlates with the score of the SF12 physical function items (SF-12phys) with R > 0.3;
3. R hypotheses 1 > R hypotheses 2;
4. The score of the MFPDI- pain items (MFPDI-p) correlates with the score of the FFI- 5pts pain items (FFI-p) with R > 0.5;
5. The score of the MFPDI-p correlates with the score of the Pain Numeric Rating Scale (NRS-p) with R > 0.5;
6. R of the MFPDI-f - SF-12 phys > R of the MFPDI-f - the SF12 mental function items (SF-12ment);
7. R of the MFPDI-f - SF-12 phys > R of the MFPDI-p - SF-12 phys.

The construct was deemed valid if 5 out of 7 hypotheses were confirmed [25].

### Cross-cultural validity

Differential Item Functioning Analyses (DIF analyses) between NL T0 and UK NorStOP data using ordinal logistic regression based on IRT was used to test cross-cultural validity. An IRT based model does not use observed sub-scale scores but incorporates item difficulty with sub-scale score providing an estimated score of the latent trait (e.g. foot function, foot related pain); theta. A negative theta implied a low dysfunction, a positive theta more foot dysfunction. The responses to each item (dependent variables) by Dutch and UK participants with similar foot dysfunction were compared to evaluate if country of origin (independent variable) significantly affects the response [28]. An item displays DIF when patients with the same estimated theta on the sub-scale do not have the same probability of endorsing that item. There are two kinds of possible DIF: uniform and non-uniform. Uniform DIF means that in one population an item is endorsed less (or more) often at *all* values of the construct, compared to the other population. Non-uniform DIF means that in one population an item is endorsed less (or more) often at *some* values of the construct, but more (or less) often at *other* values of the construct, compared to the other population. Non-uniform and uniform DIF are comparable to respectively effect modification and confounding in epidemiology. A significant effect was present if more then 2% of the variance (R^2^) was due to country of origin. A uni-dimensional construct could only be tested if the sub-scale consists of at least five items, sub-scales with less items were not analysed [29].

#### Responsiveness

In order to evaluate if the MFPDI was responsive to change a construct approach was chosen by absence of a gold standard. Change scores between the NL T0 and NL T3 data were calculated. The following seven hypotheses comparing the change scores of the functional limitation and pain sub-scales of the MFPDI to the change scores of FFI-5pt, SF12 and the NRS and to the GPE questions using Pearsons correlation (p < 0.05):

1. The change score of the MFPDI- function items (MFPDI-f) correlates with the change score of the FFI- 5pts function items (FFI-f) with R > 0.5;
2. The change score of the MFPDI-f correlates with the change score of the SF12 physical function items (SF-12 phys) with R > 0.3;
3. The change score of the MFPDI-f correlates with the GPE-function question with R > 0.5;
4. R hypotheses 1 > R hypotheses 2;
5. The change score of the MFPDI- pain items (MFPDI-p) correlates with the change score of the FFI- 5pts pain items (FFI-p) with R > 0.5;
6. The change score of the MFPDI-p correlates with the change score of the Pain Numeric Rating Scale (NRS-p) with R > 0.5;
7. The change score of the MFPDI-p correlates with the GPE-pain question with R > 0.5;

The questionnaire was deemed responsive when 5 out of 7 hypotheses were confirmed [25].

#### Interpretability

A MIC was established using the visual anchor-based MIC distribution method [30]. GPE scores were used to calculate sensitivity and specificity at each possible cut-off point for the changes in MFPDI scores between NL T0 and NL T3; the MIC is the change score for which the sum of proportion of misclassification is smallest (i.e. (1-sensitivity) + (1-specificity)). A MIC was only calculated if responsiveness hypotheses three and seven were confirmed [30].

The SDC was calculated using the SEM_agreement_ (equation 3)
3

The calculated MIC has to be bigger than the SDC in order for important change to be distinguishable from measurement error in individual patients.

Lastly the presence of a floor or ceiling effect was evaluated within the NL T0 data. If 15% of the candidates obtained the minimum or maximum sub-scale score a respectively floor or ceiling effect was present [27].

#### Software

CFA and EFA were established using M + version 6.11. The ‘lordif’ package in R was used for analysis of cross-cultural validity [28]. SPSS (IBM SPSS Statistics for Windows, Version 20.0. Armonk, NY: IBM Corp.) was used to evaluate the remaining measurement properties. All measurement properties were evaluated for every uni-dimensional sub-scale derived from the previous mentioned EFA and CFA.

## Results

### Translation

Comparison of Dutch version of the questionnaire to the original English version demonstrated four differences that needed attention. The original item one states "I avoid walking outside *at all*", the "at all" was missing in the Dutch and back translated version, and therefore ‘geheel’ was added to the statement. Item 2 ("I avoid walking *long* distances") was re-translated to: "I avoid walking *longer* distances" ("Ik vermijd het lopen over *langere* afstand"). After back translation this was changed into: "Ik vermijd het lopen over *lange* afstanden" which back translates better into the original version. The original item 10 states: "I *still* do everything but with more pain or discomfort". After back translation "still" was missing; "nog steeds" was added to the Dutch version. And finally questions 12 and 13: there is no literal Dutch word for "self-conscious"; "negatief bewust" was our first choice which means: "negatively aware". After discussion during the back translation we opted for: "verlegen" ("shy") which in Dutch language is closer to the original although it will be impossible to get an exact translation. After pilot testing (n = 10) no more changes where made to the Dutch version of the MFPDI.

### Participants

The characteristics of the participants at screening, baseline and three months for the Dutch (NL Ts, NL T0 and NL T3) and the UK (UK NOrStOP; n = 370) participants are presented in Table 1. The UK participants scored significantly higher (p < 0.001) compared to the Dutch participants on the severity of foot related disability and foot pain (higher score indicates more disability or pain). The Dutch participants scored significantly lower on both the physical and mental components of the SF12 questionnaire (lower score indicates lower well-being). Baseline characteristics of participants remaining in the NL T3 (n = 178) and NL Ts (n = 195) do not differ significantly from those in the NL T0 (n = 205). Ten NL Ts were not used since the screening form was completed on the same or previous day as completion of NL T0.

**Table 1:** **Participant characteristics and item responses**

		NL Ts (n = 195)	NL T0 (n = 205)	NL T3 (n = 169)	UK NOrStOP (n = 370)
Age: mean (SD)		-	64.1 (9.4)	-	65.3 (9.4)
Gender (% Female)		-	77.6%	-	*63%
MFPDI sub-scale scores: mean (SD)	Functional limitation	5.9 (3.8)	6.2 (4.2)	5.0 (4.4)	**9.7 (5.8)
	Pain	5.1 (2.0)	4.8 (2.4)	3.6 (2.5)	***5.23 (2.6)
	Appearance	3.6 (1.5)	1.0 (1.2)	0.9 (1.2)	**1.3 (1.4)
General Health mean (SD)	SF 12 physical wellbeing	-	41.8 (6.8)	46.9 (2.4)	**47.5 (11.6)
	SF 12 mental wellbeing	-	30.8 (9.7)	33.8 (4.6)	**36.0 (11.9)

-Data was not assessed or not applicable at this point in time.

*Significant difference p < 0.001 Chi square test comparing UK NOrStOP to NL T0.

**Significant difference p < 0.001 T-test comparing UK NOrStOP to NL T0.

***Significant difference p < 0.05 T-test comparing UK NOrStOP to NL T0.

### Missing values

The highest percentage of missing values per item of the MFPDI is 2.4% (items 2, 13, 16). Combining the UK and Dutch data for the DIF analyses for the cross cultural validity item 3 had the highest percentage of missing values: 2.8%. One participant in the UK data had more than 50% missing values and this participant was excluded from further analyses.

### Factor analysis

Using EFA three sub-scales were found in NL T0 data: A foot function scale (items 1-9), a pain scale (items 10, 14-17) and a perception scale (items 11-13). Our factor structure differed from the previously reported factor structure [7, 11, 12] by the location of item 11 (‘I get irritable when my feet hurt’). In our analysis it was included in the perception scale whereas in previous studies it was included in the functional limitations scale. Our factor structure demonstrated the best fit in both data sets (Table 2).

**Table 2:** **Results of measurement properties related to: validity**

Measurement property	Research question	Method	Dataset(s)	Results	Interpretation
Structural validity (Factor analysis)	Do the Dutch MFPDI and the NorStOP MFPDI consist of the same factor structure (sub-scales) as the original?	ECA	NL T0	Factors found:	A slightly different factor structure fitted better in both data sets than the previously reported factor structure.
		CFA	n = 205	Functional construct: 1-9	
			UK NorStOP	Pain construct: 10, 14-17	
			n = 365	Perception construct: 11-13	
				Previously reported factor structure:	The previously reported factor structure fitted acceptable in the UK dataset, but not in the Dutch dataset.
				RMSEA NL: 0.07 UK:0.05	
				CFI NL: 0.94 UK: 0.98	
				TLI NL: 0.93 UK: 0.98	
				Factor structure found in this study:	
				RMSEA NL: 0.06 UK:0.04	
				CFI NL: 0.96 UK: 0.99	
				TLI NL: 0.96 UK: 0.99	
Cross cultural validity	Assuming a similar ‘true value’ for foot related disability, does the Dutch population has the same probability of endorsing a certain response option on the items of the MFPDI as compared to the UK population?	DIF analysis using ordinal regression analyses.	NL T0	Foot function sub-scales: no DIF.	Assuming a similar ‘true value’ for foot related disability, the Dutch population has a higher probability of endorsing the response option "none of the time’" or "on some days" on item 17 as compared to the UK population
			n = 205	Foot pain sub-scale:	
			UK NorStOP	Item 17 has DIF;	
			n = 365	R^2^ = 0.048	
				Theta for transition score 0 to 1:	
				NL = -1.38, UK = -0.29	
Construct validity (hypotheses testing)	Does the MFPDI relate to other instruments as expected, based on the study of Garrow et al. [7]	Pearsson Correlation*	Comparator instruments:	Pearsson Correlations:	Construct validity is accepted; all hypotheses were confirmed.
		Testing 7 *a priori* defined hypotheses:			
		1. Correlation MFPDI-f and FFI-f (R > 0.5).	NL T0	1. R 0.66 (p < 0.000)*	
		2. Correlation MFPDI-f and SF12-phys (R > 0.3).	n = 205	2. R 0.31 (p < 0.000)*	
		3. R hypotheses 1 > R hypotheses 2		3. R 0.66 > 0.0.31*	
		4. Correlation MFPDI-p and FFI-p (R > 0.5)		4. R 0.60 (p < 0.000)*	
		5. Correlation MFPDI-p and pain NRS (R > 0.5)		5. R 0.53 (p < 0.000)*	
		6. R MFPDI-f - SF-12 phys > R MFPDI-f - SF12 ment		6. R 0.31 > R 0.14 (p = 0.045)*	
		7. R MFPDI-f - SF-12 phys > R MFPDI-p - SF-12 phys		7. R 0.31 > R 0.22 (p = 0.002)*	
				*A priori defined hypothesis confirmed	

*MFPDI-f = MFPDI- function items, FFI-f = FFI- 5pts function items, SF-12 phys = SF12 physical function items, GPE-f = GPE-function question, MFPDI-p = MFPDI- pain items, FFI-p = FFI- 5pts pain items, NRS-p = Pain Numeric Rating Scale, GPE-p = GPE-pain.

#### Reliability

**Internal consistency** The functional limitation sub-scale was deemed to be internal consistent (Cronbach’s α > 0.7). The internal consistency of the pain and perception sub-scales were moderate (Table 3).

**Table 3:** **Results of measurement properties related to: reliability**

Measurement property	Research question	Method	Dataset(s)	Results	Interpretation
Internal consistency	Do the items within the (uni-dimensional) sub-scales correlate highly?	Cronbach’s α (per sub-scale)	NL T0	Function: 0.84	The foot function scale is internally consistent (>0.7). Internal consistency of the other sub-scales is moderate.
			n = 205	Pain: 0.67	
				Perception: 0.61	
Test-retest reliability	Does the Dutch MFPDI produce similar results when completed repeatedly within an interval of two weeks?	ICC absolute agreement (per sub-scale)	NL Ts and NL T0	Function: 0.69	The test-retest reliability of the function scale is nearly acceptable. Neither of the other scales is reliable.
			n = 195	Pain: 0.49	
				Perception: 0.10	
Measurement error (agreement)	Which part of the variance is due to measurement error?	SEM (per sub-scale)	NL Ts and NL T0	Function: 2.2	There are no set guidelines of what is acceptable for the magnitude of SEM. Function scale SEM of total score is: 12%, pain scale: 16%, perception: 36%
			n = 195	Pain: 1.6	
				Perception: 2.1	

**Test-retest reliability** None of the ICC values reached the generally accepted limit of 0.7 although the ICC for the foot function sub-scale was 0.69 (Table 3).

**Measurement error** The SEM of the perception sub-scale was large (36% of the maximum scale score). Both the foot function and the foot pain scale contained a smaller SEM; respectively 12% and 16% of the maximum scale score.

#### Validity

**Construct validity** All *a priori* stated hypotheses about correlations of the MFPDI function and pain scales to the FFI-5pt, SF12 and NRS were confirmed (Table 2).

**Cross cultural validity** Due to the limited amount of items (<5) in the perception sub-scale, DIF analysis was not performed on this sub-scale. DIF analysis on the foot function sub-scale showed that there was no DIF between the UK and a Dutch population (Table 2). Item 17 in the foot pain sub-scale ("I get shooting pain in my feet") showed uniform DIF. Having a similar level of foot pain (theta), the Dutch population has a higher probability of answering this item with: "none of the time" or "on some days" than the UK population.

#### Responsiveness

One of the 7 *a priori* stated hypotheses about correlations between different change scores (change score = the difference between the scale score at NL T0 and NL T3) was confirmed (Table 4). Neither the function scale nor the pain scale change scores correlated to the corresponding GPE questions at an acceptable level (respectively R = 0.46 and R = 0.47). These correlations were considered too low to calculate a MIC.

**Table 4:** **Results of measurement properties related to: responsiveness and interpretability**

Measurement property	Research question	Method	Dataset(s)	Results	Interpretation
Responsiveness	Do change scores on the MFPDI relate to change scores on other instruments as expected?	Pearsson Correlation*	Comparator instruments: FFI, NRS, SF12phys, GPEpain and GPEfunction		The responsiveness of the MFPDI is moderate; only 1 out of 7 hypotheses was confirmed and the correlation with the GPE question is < 0.2.
		Testing 7 a priori defined hypotheses:			
		1. Correlation change MFPDI-f and FFI-f (R > 0.5)	T0 and measurement after 3 months (NL T3)	1. R 0.31 (p < 0.000)	
		2. Correlation change MFPDI-f and SF12 phys (R > 0.5)	n = 178	2. R 0.03 (p = 0.747)	
		3. Correlation change MFPDI-f and GPE-f (R > 0.5)		3. R -0.46 (p < 0.000)	
		4. R hypothesis 1 > R hypothesis 2.		4. R 0.31 > R 0.03*	
		5. Correlation change MFPDI-p and FFI-p (R > 0.5)		5. R 0.37 (p < 0.000)	
		6. Correlation change MFPDI-p and pain NRS (R > 0.5)		6. R 0.42 (p < 0.000)	
		7. Correlation change MFPDI-p and GPE-p (R > 0.5)		7. R -0.47 (p < 0.000)	
				*A priori defined hypothesis	
Interpretability	What is the Minimal Important change (MIC)?	MIC: smallest cut-off change score (1-sensitivity)+	MIC: NL T0,	The correlation coefficient between the GPE and change score is too low to calculate a MIC (R < 0.5).	The MFPDI is not responsive enough to calculate a MIC.
			NL T3		
		(1-specificity)	n = 178		
	What is the Smallest Detectable Change (SDC)? And is the MIC higher than the SDC?	SDC	SDC: NL T0 n = 205	SDC:	The SDC for the perception sub-scale is too large; the SDC is equal to the maximum possible score.
				Function: 6.1 (min-max score: 0–18)	
				Pain: 4.4 (0–10)	
				Perception: 5.8 (0–6)	
	Is a floor and or ceiling effect present?	Floor/ceiling effect: % of participants who scored the two lowest possible scores (0 or1) per sub-scale.	Floor/ceiling effect: NL T0 n = 205	Floor/ceiling effects:	The perception sub-scale exhibits a large floor effect.
				Function: 8.8%	
				Pain: 7.4%	
				Perception: 76.5%	

*MFPDI-f = MFPDI- function items, FFI-f = FFI- 5pts function items, SF-12 phys = SF12 physical function items, GPE-f = GPE-function question, MFPDI-p = MFPDI- pain items, FFI-p = FFI- 5pts pain items, NRS-p = Pain Numeric Rating Scale, GPE-p = GPE-pain.

#### Interpretability

The perception sub-scale showed an extreme floor effect; 86.2% of the participants obtained the minimum possible scale score. The other sub-scales do not exhibit a floor or ceiling effect (Table 4).

The SDC of the perception sub-scale is as large as the maximum obtainable score of 6 points (Table 4). Due to the inability to calculate a MIC, comparison of SDC and MIC is not possible.

## Discussion

Evaluation of the MFPDI measurement properties produced new useful information. Of the 3 uni-dimensional sub-scales found in the NL T0 data only the functional limitation sub-scale has an acceptable level of reliability. Scores from the Dutch and UK version can be interpreted similarly; only the pain subscale possesses one DIF item but the differences in item responses and thus sub-scale responses are smaller than the measurement error. The responsiveness of the questionnaire is moderate.

Several studies have investigated the presence of sub-scales within the MFPDI using either EFA and/or CFA. Although differences have been found in the number of sub-scales: 2 [8], 3 [12] or 4 [7, 10]; close inspection shows several similarities between the different outcomes. The main difference between the various factor structures is the placement of item 11 ("I get irritable when my feet hurt."). Previous analyses have placed this item in the foot function scale [7, 12], in the foot pain scale [10] and in a combined pain and appearance scale [8]. In our opinion item 11 is related to emotion due to foot pain and therefore should not be part of the foot function scale. In our data it fits best with items 12 and 13 which were previously labelled as appearance items. These two items are actually about feeling self-conscious about the appearance of feet and shoes and do not mention the appearance itself of feet and shoes. Adding item 11 to these two and naming it "perception scale" adds to the face-validity of the scale in our opinion.

### Reliability

Although internal consistency has been evaluated in every MFPDI validation study published [7–12], only two studies evaluated the IC per individual sub-scale [8, 12]. Both studies found acceptable internal consistencies (>0.7) for all tested sub-scales in contrast to our findings. Our findings suggest that the functional scale is internally consistent (0.84), the pain scale is just below acceptable (0.67) and the internal consistency of the perception sub-scale is moderate (0.60). The differences between our results and those found by Cook et al. [8] (IC of the pain and appearance scale: 0.75) could be explained by the differences in the number of items; respectively 3 and 7 items. The test-retest reliability of the foot function sub-scale is almost acceptable (ICC = 0.69) but the reliability of the pain sub-scale is moderate (0.49) and the reliability of the perception sub-scale is poor (0.10). All participants scored at least one item of the entire MFPDI as: "on most/every day(s)" and our findings are consistent with those reported by Roddy et al. [12]. A measurement error has not been established before. Our findings suggest that the SEM for the perception scale is too large; 36% of the possible maximum score.

### Validity

The UK and Dutch participants differ on baseline on the amount of foot related pain and dysfunction. However, these differences are not of influence on the cross-cultural validity analyses because item responses of UK and Dutch patients with similar ‘true values’ are compared to each other.

Assessment of the cross cultural validity by means of DIF analysis showed that Dutch and UK participants with a similar level of foot dysfunction complete the foot function sub-scale in a similar manner. A small difference was found in the completion of the foot pain sub-scale. Assuming a similar ‘true value’ for foot related disability, the Dutch population has a higher probability of endorsing the response option "none of the time’" or "on some days" on item 17 as compared to the UK population. We expect that the DIF of item 17 is due to the difference in location of the foot pain. The UK population was included if they had pain in any part of the foot whereas the Dutch population was selected when having pain in the forefoot or toes. It is plausible that the characteristics of forefoot pain might differ from the characteristics of general foot pain explaining the difference between the Dutch and UK population.

The seven *a priori* stated hypotheses were all confirmed and thus the foot function and the pain scales seem to be valid. Due to absence of a comparator instruments the validity of the perception scale has not been assessed. In a previous study by Menz et al. [10] the MFPDI scores were compared to other patient reported outcomes like the GADS depression sub-scale and the SF-36 mental health and general health sub-scale. These correlation coefficients were lower than those in our results; their highest correlation was R = 0.34. This is probably due to the fact that the comparator instruments differ in both studies and that the instruments used in this study are more foot related. Even though the construct validity is acceptable based on these findings, we are hesitant about the face validity of the foot pain scale. Previous authors have described this scale as measuring *pain intensity* [7, 11, 12]. Examining the items in this scale it is striking that none of the items actually posses a reference to pain intensity. The items mainly ask about *when* the foot pain is worse and about the *kind* of pain. This scale also contains several opposing statements. Item 15 states: "My feet are worse in the morning" whilst item 16 states: "My feet are more painful in the evening". Items 14 and 17 are somewhat opposing as well: "I have constant pain in my feet" versus I have shooting pain in my feet". We are hesitant to use this sub-scale if *pain intensity* is the construct of interest.

### Responsiveness and interpretation

Neither responsiveness nor a MIC have previously been established. Comparing the change scores of the foot function and the pain sub-scale to changes in comparator instruments like the foot function index-5 pt (foot related activities and pain sub-scales), the SF12 physical component and the pain NRS, only moderate correlations were found. But most importantly, the correlation between the change scores and anchor question (GPE question) were only moderate (R = 0.43-0.47). We considered these correlation coefficients too low (below 0.5) to calculate a MIC. There are multiple possible explanations for the moderate responsiveness. With regard to the entire questionnaire, it could be that the response options (none of the time, on some days and on most/every day(s)) are too widely spaced to be able to measure small changes over time. With regard to the foot function sub-scale; the questionnaire uses items that clearly begin with: ‘because of the pain in my feet…’. Even so, the activities stated could very likely be influenced by other variables like loss of muscle strength or pain in other joints, especially within an older population. It could possibly be hard to distinguish the inability to do something because of foot pain or other pains and therefore it might not respond to change if only one of the problems improves. The SDC is calculated based on the SEM and so the perception scale has an extreme high SDC; 5.8 points on a scale that has a 6 point maximum. This sub-scale also has a large floor effect; 76.5% of the participants have a score of 0 or 1 point. Combining these two findings, it will be improbable to find a change within this scale.

The main strength of this study is that the full array of measurement properties based on CTT has been evaluated. Nevertheless, the results of this study should be interpreted in light of its limitations. The group of participants used for this evaluation of the MFPDI (NL T0) is very homogeneous; >50 years of age, visited their GP with forefoot pain not due to rheumatoid arthritis or skin lesions and no diabetic neuropathy. Particularly the attribute of having forefoot pain is different compared to other studies. Even though our results do not seem to differ a lot from previously published work, measurement properties like responsiveness, MIC, SDC and SEM have not been assessed before. And thus it is unsure if, for instance, the moderate responsiveness the MFPDI holds for populations with other kinds of foot pain as well. Another limitation is that the power in this study was insufficient to assess DIF for variables like to age, gender and location of foot pain.

Although foot pain in general is more common in woman than in man [2, 3]. The percentage of women in this study (77.6%) differ from both gender distributions reported by Garrow et al. [2] (59.6%) or the UK NOrStOP data (63%). These studies contain people with foot pain in general. It could be that women are, more so than men, predisposed to have forefoot problems, compared to pain anywhere in the foot. Nevertheless, most outcomes in this study are comparable to those of other studies [9–12]. We therefore assume that the difference in gender distribution does not affect the outcome of this study.

## Conclusion

Results using the Dutch version of the MFPDI are not different from those using the English version since the function scale has no DIF and the pain scale only a small amount. Due to the limited reliability, moderate responsiveness, floor effect and large measurement error of both the pain and most of all the perception scale, found in our study and other studies it is advisable that the items in these scales (item 10-17) are used with extreme caution. Quantifying pain by the use of a NRS or VAS can be an alternative. The reliability of the function scale (items 1-9) is acceptable as is its construct validity. The moderate responsiveness of the MFPDI function scale should be taken into consideration when using it in a longitudinal study. It is unclear if these results would be different for participants with foot pain other than the forefoot or if the results are influenced by problems in the entire lower extremity.

## Abbreviations

CFA: Confirmatory factor analysis
CTT: Classical Test Theory
DIF: Differential item functioning
EFA: Exploratory factor analysis
FFI: Foot function index
GPE: Global perceived effect (also known as anchor question)
IC: Internal consistency
ICC: Intraclass correlation
IRT: Item response theory
MFPDI: Manchester Foot Pain and Disability Index
MIC: Minimal important change
NL Ts: T0, T3, Dutch datasets respectively just prior to baseline, baseline and after 3 months
NRS: Numeric rating scale
SEM: Standard error of measurement
SDC: Smallest detectable change.
